# AVNRT captured by Apple Watch Series 4: Can the Apple watch be used as an event monitor?

**DOI:** 10.1111/anec.12742

**Published:** 2020-01-29

**Authors:** Hani Siddeek, Kaylee Fisher, Sandra McMakin, John L. Bass, Daniel Cortez

**Affiliations:** ^1^ Department of Pediatrics University of Minnesota/Masonic Children's Hospital Minneapolis MN USA; ^2^ Park Center Minneapolis MN USA; ^3^ Fairview Health Systems Minneapolis MN USA; ^4^ Clinical Sciences Lunds Universitet Lund Sweden

**Keywords:** ablation, Apple Watch, AVNRT

## Abstract

Wrist‐worn devices are popular for heart rate monitoring, including use of photoplethysmography. The Apple Watch series 4 can identify atrial fibrillation. We describe a case of identification re‐entrant supraventricular tachycardia not identified by outpatient rhythm monitoring, however, was identified by the Apple Watch series 4, which lead to electrophysiology study and successful ablation of atrioventricular nodal re‐entrant tachycardia.

## 1 CASE REPORT

Wrist‐worn devices have surged in popularity offering heart rate (HR) monitoring using photoplethysmography that noninvasively detects HR utilizing optical properties of vascular tissue, usually via LEDs (Ip, 2019). The Apple Watch Series 4 has the capability of recording a 30‐s single channel ECG and could provide a means of documenting rhythm during symptoms in some patients.

## 2 CASE

**Figure 1:**
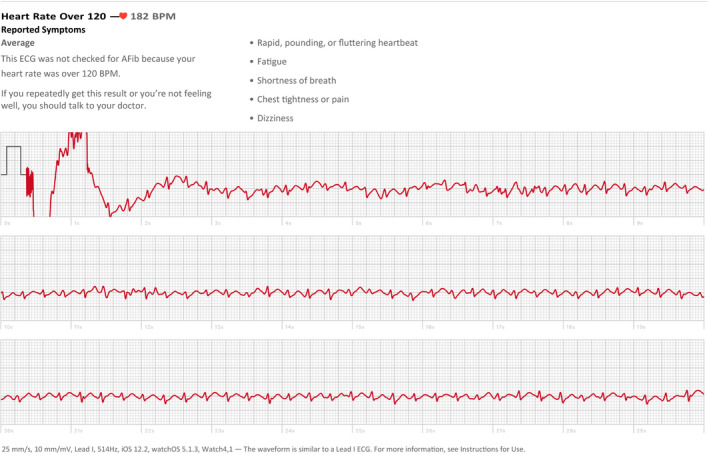
Apple Watch recording of short RP tachycardia with rate of 182 bpm. Note retrograde. P waves noted

A 16‐year‐old female had atrioventricular nodal reentry tachycardia (AVNRT) that was captured on an Apple Watch Series 4. She began having palpitations 3 years before presentation, with shortness of breath, decreased vision, and headache. Episodes were associated with exercise and lasted 30–60 min. She captured an episode on the Apple watch that demonstrated tachycardia at a rate of about 225 bpm, but with significant artifact. She wore a Zio patch monitor for 2 weeks but was asymptomatic with no arrhythmias. Subsequently, she submitted an ECG recording from her Apple Watch Series 4 (Figure 1) from a symptomatic event that revealed a short RP tachycardia suggesting AVNRT.

An electrophysiology study showed dual AV nodal pathways at baseline. On Isuprel, she had inducible short RP tachycardia with cycle length of 288 ms and V‐A interval of 58 ms. Slow junctional rhythm was induced within 3 s of radiofrequency ablation at the area of the slow pathway. Two ablation lesions were placed. Neither a slow pathway nor inducible AVNRT on or off of Isuprel was noted afterward. At 1‐week follow‐up, she had no further palpitations and a normal PR interval on electrocardiogram. She remained asymptomatic at 4‐month follow‐up.

## 3 DISCUSSION

The Apple Watch Series 4 uses two electrodes to generate a single lead ECG up to 30 s, using an iPhone and Health App. The ECG can be stored as a pdf and transmitted to a physician. Traditional ambulatory ECG monitors (i.e., adhesive wired electrodes and patch‐based Holter monitors) are utilized for continuous rhythm monitoring and are accurate in detecting abnormal rhythms. These monitors offer patient‐activated intermittent nonlooping ambulatory ECG monitoring, but compliance is not reliable. The Apple Watch Series 4 does not allow continuous recording of rhythm, but can record, store, and transfer 30‐s single channel ECG recordings (Ip, 2019). A criticism is that only one lead can be recorded (Frisch, 2019). The device is also relatively expensive.

The Apple Watch Series 4 is becoming popular and gives the general public more access to ambulatory ECG monitoring and may be a viable option for rhythm monitoring.

## 4 CONCLUSION

The Apple Watch Series 4 provides a single lead (lead I) monitoring device that, when correlated with symptoms, may help identify arrhythmias. In our case, symptomatic atrioventricular nodal re‐entrant tachycardia was identified by the Apple Watch, and subsequent ablation of AVNRT was successful.

## CONFLICTS OF INTEREST

The Authors declare that they have no conflicts of interest with the contents of this atricle.

## AUTHOR CONTRIBUTIONS

All authors reviewed and approved the manuscript. Directed this study: Daniel Cortez. Collected patient data: Hani Siddeek, Kaylee Fisher. Wrote the main manuscript: Hani Siddeedk, Daniel Cortez. Gave suggestions for the study: John Bass.

## ETHICS

The study was approved by the ethics committee at the University of Minnesota. The study was conducted in accordance with the principles of the Declaration of Helsinki.
